# Pancreatic stump closure techniques and pancreatic fistula formation after distal pancreatectomy: Meta-analysis and single-center experience

**DOI:** 10.1371/journal.pone.0197553

**Published:** 2018-06-13

**Authors:** Elke Tieftrunk, Ihsan Ekin Demir, Stephan Schorn, Mine Sargut, Florian Scheufele, Lenika Calavrezos, Rebekka Schirren, Helmut Friess, Güralp O. Ceyhan

**Affiliations:** Department of Surgery, Klinikum rechts der Isar, Technical University Munich, Munich, Germany; Universita degli Studi di Verona, ITALY

## Abstract

**Background:**

Pancreatic fistula/PF is the most frequent and feared complication after distal pancreatectomy/DP. However, the safest technique of pancreatic stump closure remains an ongoing debate. Here, we aimed to compare the safety of different pancreatic stump closure techniques for preventing PF during DP.

**Methods:**

We performed a PRISMA-based meta-analysis of all relevant studies that compared at least two techniques of stump closure during DP with regard to PF rates/PFR. We further performed a retrospective analysis of our institutional PFR in correlation with stump closure techniques.

**Results:**

8301 studies were initially identified. From these, ten randomized controlled trials/RCTs, eleven prospective and 59 retrospective studies were eligible. Stapler closure (26%vs.31%, OR:0.73, *p* = 0.02), combination of stapler and suture (30%vs.33%, OR:0.70, *p* = 0.05), or stump anastomosis (14%vs.28%, OR:0.51, *p* = 0.02) were associated with lower PFR than suture closure alone. Spleen preservation/splenectomy, or laparoscopic/open DP, TachoSil^®^, fibrin-like glue-application, or bioabsorbable-stapler-reinforcements (Seamguard^®^) did not influence PFR after DP. In contrast, autologous patches (falciform ligament/seromuscular patches) resulted in lower PFR than no patch application (21.9%vs.25,8%, OR:0.60, *p* = 0.006). In our institution, the major three techniques of stump closure resulted in comparable PFR (suture:27%, stapler:29%, or combination:24%). However, selective suturing/clipping of the main pancreatic duct during pancreatic stump closure prevented severe PF (*p* = 0.02).

**Conclusion:**

After DP, stapler closure, pancreatic anastomosis, or falciform/seromuscular patches lead to lower PFR than suture closure alone. However, the differences are rather small, and further RCTs are needed to test these effects. Selective closure of the main pancreatic duct during stump closure may prevent severe PF.

## Introduction

Perioperative morbidity and mortality rates after pancreatic resection have continuously diminished over the past two decades in parallel with concentration of pancreatic resections in “pancreatic surgical centers” [1, 2]. However, even in high-volume centers, pancreatic fistula (PF) remains a frequent pancreas-specific complication after pancreatic resection, reaching a prevalence of approximately 30% after distal pancreatectomy (DP) [3]. Although some factors such as unligated main pancreatic duct [4], high body-mass index [5], or intraoperative blood loss [5] have been recognized as risk factors for developing PF after DP, no single surgical technique or innovation has yet been reported to considerably reduce PF rates after DP in a prospective setting. The persistently high PF rates after DP worldwide indicate that our understanding of the pathophysiology of PF after DP is still insufficient. Furthermore, there seems to be discrepancies in the reporting and interpretation of the results from the increasing number of studies that compared different closure techniques for the pancreatic stump after DP.

So far, stump closure after DP has been reported to be mainly performed by six different techniques: 1) manual/hand sutures on the stump to close the draining pancreatic duct [3], 2) stapler-based transsection and concomitant closure of the stump [3], 3) combination of stapler-based resection with manual sutures along the stapler line [4], 4) pancreatico-enteric or -gastric anastomosis [6], 5) application of fibrin/coagulation factor-like bio-sealants [7], 6) placement of autologous patches like falciform ligament [8] or seromuscular seals [9] on the pancreatic stump. In addition to these techniques, laparoscopic DP represents a rather novel technical aspect that has not yet been sufficiently compared to open DP with regard to PF frequency [10]. In recent years, some of these techniques have been compared in the framework of few randomized trials [3, 9], and the evidence provided by retrospective case series does not always overlap with the outcome of such trials [11]. Furthermore, there is a great discrepancy in the PF rates after DP, ranging between 12% and 51% [12, 13], and also major differences in the stump closure techniques in various pancreatic centers worldwide.

In the present study, we performed a systematic review and meta-analysis of the PF rates reported to occur with the described six different stump closure techniques after DP. Furthermore, we compared the PF outcome of these different stump closure techniques after DP at our department and thereby provided a comparative overview of our single-center experience.

## Methods

The study was in line with the ethics requirements and was approved by the Ethics Committtee of the Technical University Munich (Nr. 30/17s).

### Search methodology & Data extraction

To perform the meta-analysis, we conformed to the Preferred Reporting Items for Systematic review and Meta-Analysis (PRISMA) guidelines [14, 15] (S1 Checklist). Pubmed, Cochrane library, Ovid and Google Scholar were systematically searched for the terms “distal pancreatectomy”, “left pancreatectomy”, “distal pancreatic resection”, “left pancreatic resection”, “pancreatic fistula”, “fistula” and “leak” for studies published until the end of December 2015 with restriction to articles in English. Reference sections of the included articles were additionally screened for further relevant studies. In addition, a manual search of bibliographies of related reviews was carried out for additional references. After removing duplicates, abstracts were independently screened by three reviewers (ET, IED and SS). The reviewers noted the first author, year of the study, study design and the compared techniques, and the definition of fistula in each study. Disagreement or uncertainties were resolved by the consensus of the three reviewers.

### Inclusion and exclusion criteria

The study included retrospective case series (which represent the majority of all studies performed in the field), prospective case studies, and randomized controlled trials. In all *included* studies, at least two techniques of pancreatic stump closure were compared in two groups of patients undergoing DP, and quantitative data were available on the frequency of postoperative PF. Criteria that led to study *exclusion* were lack of quantitative data on postoperative PF rates, lack of comparison of at least two techniques, analysis of resections other than DP (e.g. pancreaticoduodenectomy), inconclusive remarks on the uniformity of the applied stump closure techniques (e.g. “manual suture ± biosealant”), or impossibility to extract the odds ratio based on the reported PF frequencies.

### Assessment of evidence

The level of evidence provided by each individual study was judged based on the recommendations of the Oxford Centre for Evidence-Based Medicine, “The Oxford Levels of Evidence 2” (http://www.cebm.net/index.aspx?o=5653).

### Retrospective analysis

The PF rates of patients who underwent DP between 2007 and 2015 at the Department of Surgery, Klinikum rechts der Isar, Technische Universität München, Germany, were derived from a prospectively continued departmental database. DP was performed by seven different experienced surgeons. The PF grades were defined according to the definition of the International Study Group on Pancreatic Fistula (ISGPF) [16].

### Statistical analysis

Statistical analysis was performed using Review Manager Software (Review Manager/RevMan,Version 5.3, Copenhagen, The Nordic Cochrane Centre, The Cochrane Collaboration, 2012). The meta-analysis was performed according to the recommendations provided by the Quality of Reporting of Meta-Analyses guidelines [17]. Dichotomous data, i.e. presence or absence of PF, were analyzed using odds ratio (OR) as the summary statistic. PF rates after different closure techniques were compared in 11 different meta-analyses. The primary outcome parameter was the overall PF rate. The OR of PF from each study was weighted by the sample size and reported together with the 95 per cent confidence interval. A two-sided p-value was calculated and a level of significance of α = 0.05 was used. To compensate for heterogeneity, the Mantel-Haenszel method with a random-effects model was used in the meta-analysis to ensure more conservative ORs. Squares on the graphs correspond to the point estimates of the treatment effect of each study, and the flanking horizontal lines depict the 95 per cent confidence interval In the meta-analysis, heterogeneity between the included studies was quantified using the inconsistency statistic (I^2^), where a I^2^ greater than 50% indicates high heterogeneity. The institutional data on PFR were compared via the Chi-square test.

### Investigation of publication bias

The RevMan 5.3 software was utilized to generate a funnel plot for each meta-analysis. Publication bias could be excluded if each point is evenly or symmetrically distributed and lying within the 95 per cent confidence interval that is indicated by the virtual triangle.

## Results

**Fig 1:**
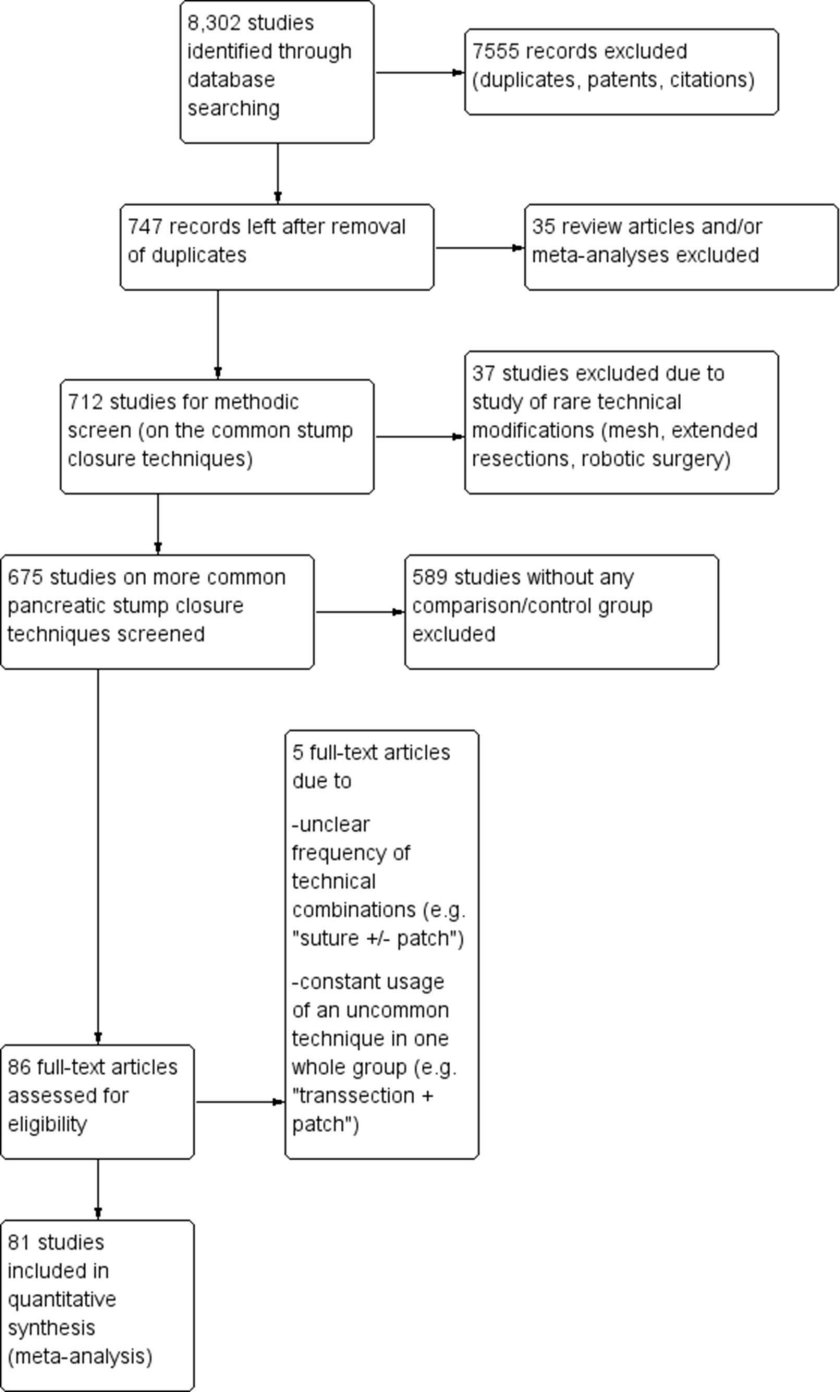
Flowchart of the systematic review and meta-analysis. The boxes to the right indicate the number of excluded studies with the specified reasons.

A total of 8,302 studies were identified after the literature search in various databases (Fig 1). After elimination of duplicates, patents, citations, or non-English articles, 747 potentially relevant studies were screened in their abstracts. From these, 35 review articles or meta-analyses were excluded. In the screening of the remaining abstracts 37 additional studies that dealt with rare technical modifications (e.g. mesh augmentation, extended resections, robotic surgery) were excluded. In 589 studies, we detected no control/comparison group, so that 86 full-text articles were available for the derivation of the quantitative data. In 5 full-text articles, the exact distribution of technical combinations was not indicated (e.g. “suture ± patch”), or they lacked quantitative data. 81 studies were therefore available for the systematic review and meta-analysis. There were 9 randomized controlled trials (RCTs) [3, 7, 9, 18–23] with evidence level 2a, 1 small RCT with evidence level 2b [24], and 59 retrospective case-control studies with evidence level 4. The pooled odds ratios (ORs) and the total number of included patients for each type of sub-analysis are depicted on S1 Table.

### Comparison of stump closure techniques

#### Stapler vs. suture

**Fig 2:**
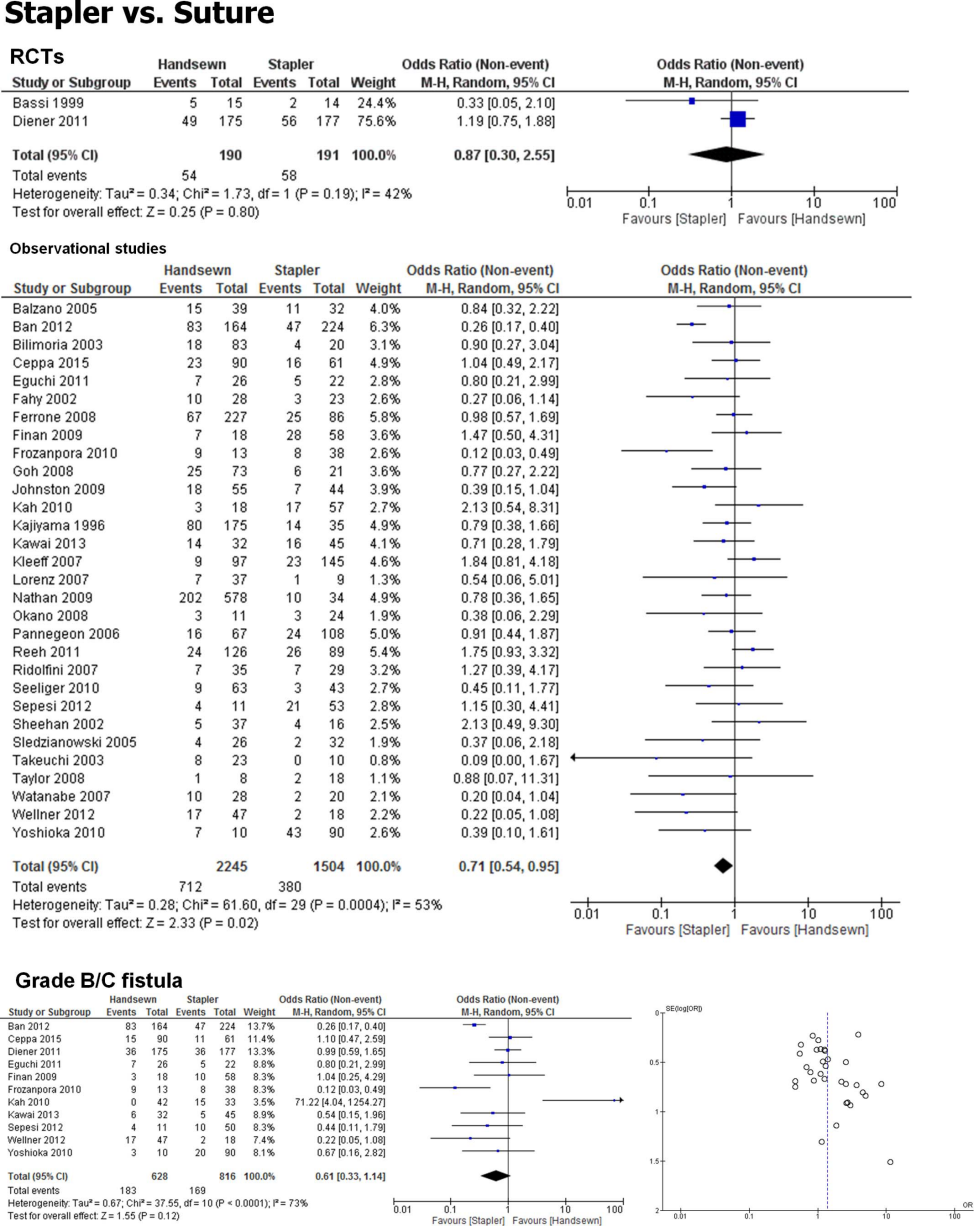
Stapler closure of the pancreatic remnant during distal pancreatectomy/DP is associated with less frequent pancreatic fistula/PF formation. Forrest plot of studies that provided quantitative data on the PF rates after handsewn/suture vs. stapler closure of the pancreatic stump during DP. 95%CI: 95% confidence interval. RCTs: randomized controlled trials. Grading of PF as B or C in the eligible studies was according to the ISGPF definition [16]. Bottom right: funnel plot of included studies.

First, the PF rate after DP was compared between stapler and handsewn (suture) closure of the pancreatic stump. In the meta-analysis of the 32 studies with a total of 4,130 patients, stapler closure was associated with a reduced PF rate after DP (OR 0.73, 0.56 to 0.95; *p* = 0.02). In the separate analysis of the 2 RCTs that included this comparison, this favorable effect of stapler vs. suture closure was not detectable anymore (OR 0.87, 0.30 to 2.55; *p* = 0.80), as also shown previously [11]. Furthermore, the comparison of clinically relevant grade B or C fistula did also not reveal any difference between the two techniques (OR 0.61, 0.33 to 1.14; *p* = 0.12, S1 and S2 Tables), The analysis of heterogeneity revealed high heterogeneity (*I*^*2*^ = 54%), and the funnel plot for the presence of potential publication bias showed an asymmetrical distribution of the studies (Fig 2), where studies that may have pointed out toward a benefit from suture seemed to be unreported.

#### Combination of stapler and suture closure

**Fig 3:**
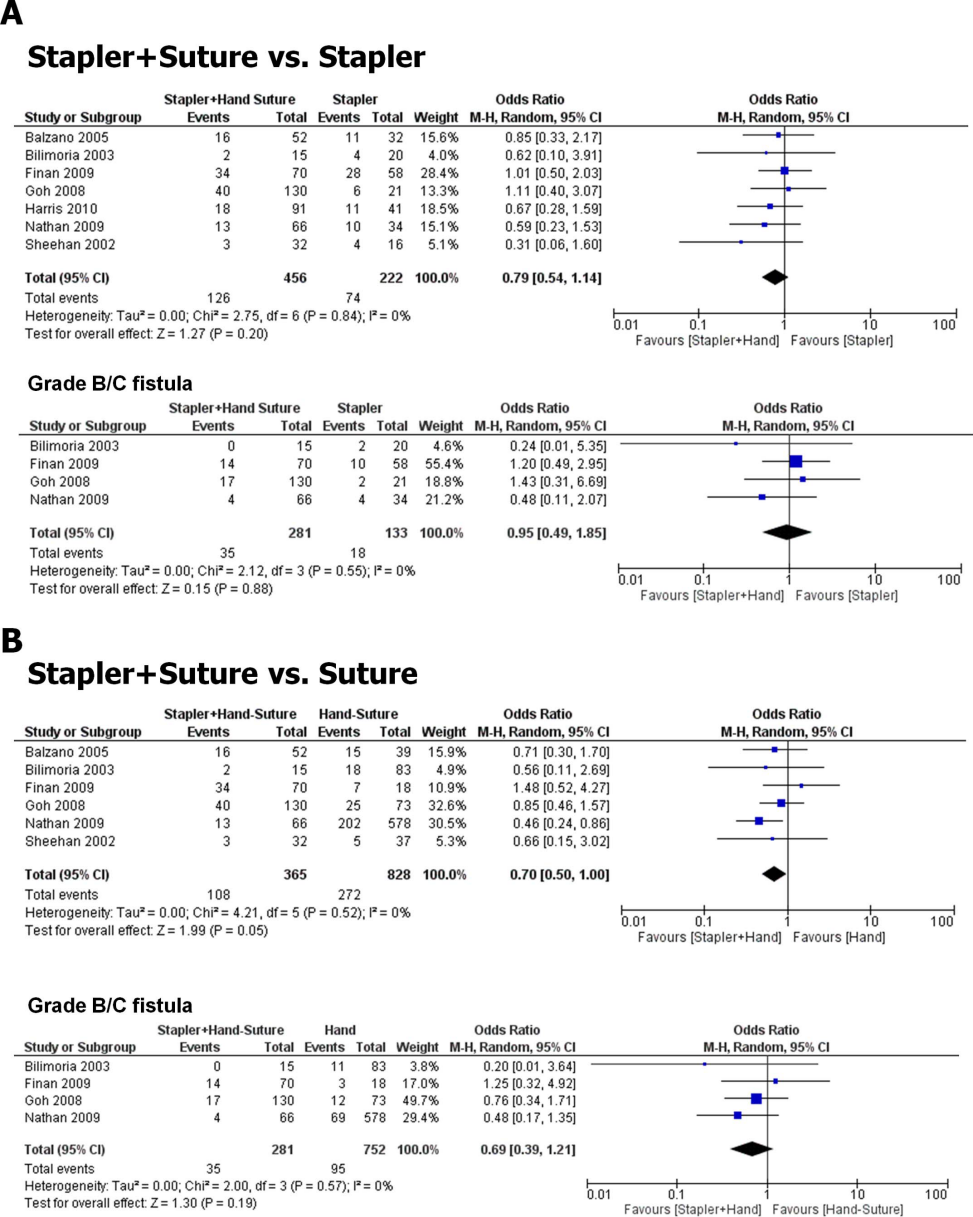
Impact of stapler and suture combination on pancreatic fistula rates during DP. **A.** Forrest plot of studies that compared the overall (upper plot) and clinically relevant (grade B/C, lower plot) PF rates after DP with either combined stapler and suture closure versus isolated stapler closure of the pancreatic stump. **B.** Forrest plot of studies that compared the overall (upper plot) and clinically relevant (grade B/C, lower plot) PF rates after combined stapler and suture closure during DP versus isolated suture of the pancreatic stump. 95%CI: 95% confidence interval.

We identified 7 retrospective studies with a total number of 1,193 patients [4, 5, 25–29] in which stapler and suture transection were combined and compared with manual sutures or stapling alone during DP (Fig 3A and 3B). Here, no difference in the overall PF rate or rate of grade B/C PF was detected in the comparison of combination closure versus stapling alone (OR 0.79, 0.54 to 1.14; *p* = 0.20, Fig 3A), whereas combination closure tended to be superior to suturing alone (OR 0.70, 0.50 to 1.00; *p* = 0.05, Fig 3B). However, this effect was not present in the comparison of clinically relevant grade B or C fistula (OR 0.69, 0.39 to 1.21; *p* = 0.19, S1 and S2 Tables). There was no heterogeneity between these studies (*I*^*2*^ = 0%). According to the Cochrane recommendations ^(^^http://handbook.cochrane.org/chapter_10/10_4_3_1_recommendations_on_testing_for_funnel_plot_asymmetry.htm^^)^, funnel plots were not generated due to the low number of included studies in this analysis.

#### Enteric or gastric anastomosis vs. suture

**Fig 4:**
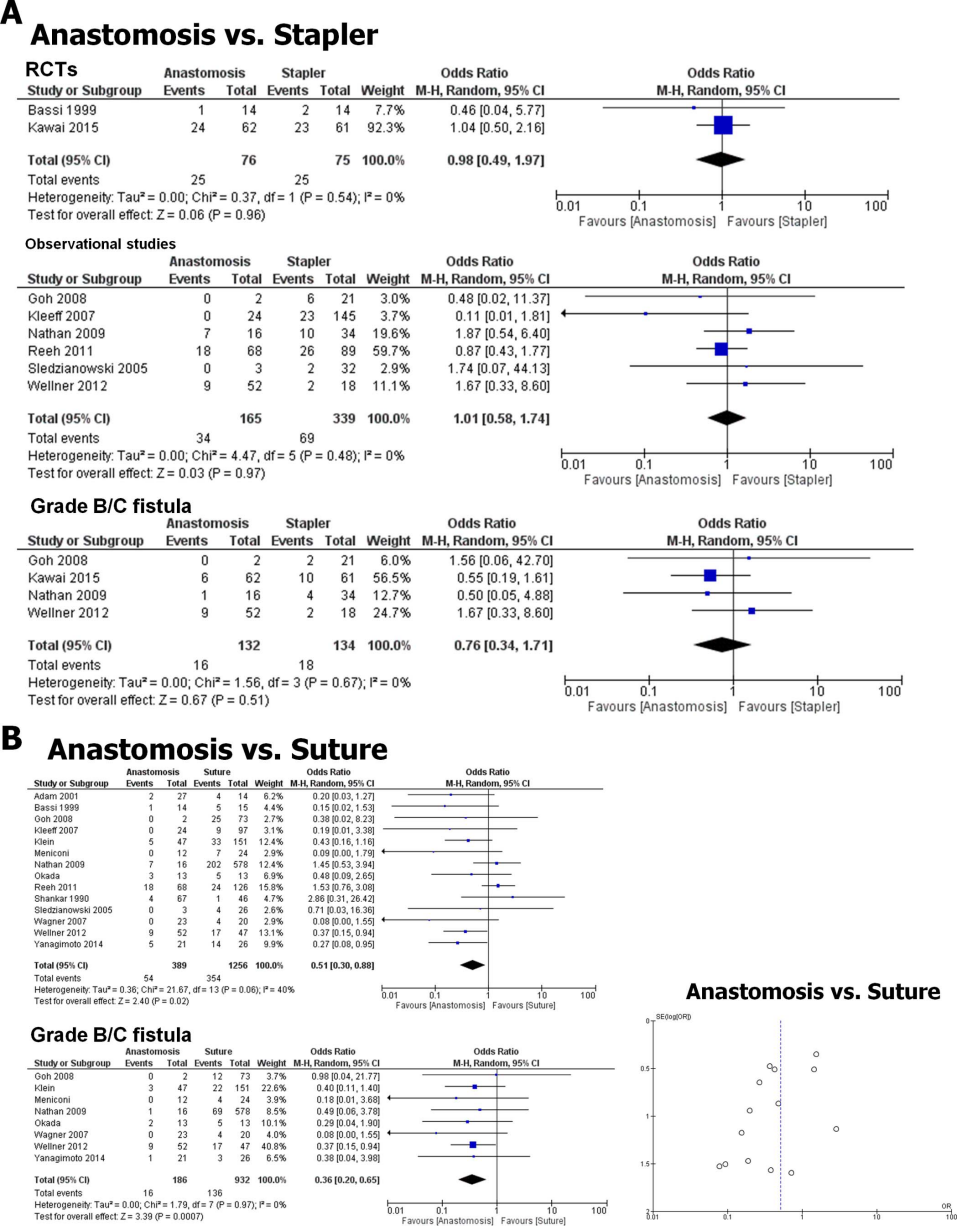
Anastomosis of the pancreatic stump during DP is superior to suture, but not to stapling, for reducing fistula rates. **A.** Forrest plot of studies that compared anastomosis (i.e. pancreatico-gastrostomy or–enterostomy) of the pancreatic stump to stapler closure with regard to overall (upper plot) and clinically relevant (grade B/C, lower plot) postoperative PF rates after DP. RCTs: randomized controlled trials. **B.** The Forrest plot of studies that compared anastomosis to suture closure of the pancreatic stump show a beneficial effect of anastomosis for reducing overall (upper plot) and clinically relevant (grade B/C, lower plot) postoperative PF rates after DP. Bottom: funnel plot of included studies in the comparison of anastomosis vs. suture with regard to PF rates after DP.

Anastomosis of the pancreatic stump (e.g. pancreatico-jejunostomy [22], pancreatico-gastrostomy [30]) has been considered as an alternative method of stump closure especially among patients with small, hardly recognizable main pancreatic duct [6]. There was one recent RCT comparing this technique to stapler closure, and this RCT did not show any benefit for anastomosis versus stapler closure [22]. We identified 8 studies (2 RCTs, 1 prospective, 5 retrospective, S1 Table), with a total number of 655 patients [5, 12, 22, 24, 28, 31–33] that compared the PF rates after pancreatic anastomosis versus stapler-based closure of the pancreatic stump (Fig 4A). Here, anastomosis closure did not reveal a reduction in the overall PF rate (OR 1.00, 0.65 to 1.53, p = 0.99) or of clinically relevant grade B/C PF rate (OR 1.01, 0.58 to 1.74, p = 0.97, S1 and S2 Tables), which was also in line with the findings of the most recent RCT by Kawai et al. [22] (Fig 4A). Accordingly, in the separate analysis of the 2 RCTs and observational studies, there was again no superiority of either technique (RCTs: OR 0.98, 0.49 to 1.97; *p* = 0.96; Observational studies: OR 1.01, 0.58 to 1.74; *p* = 0.97, Fig 4A). On the other hand, we identified 14 studies [5, 6, 12, 24, 28, 30–38] (1 RCT [24], 3 prospective, 10 retrospective studies) with a total number of 1,645 patients that compared the overall PF rates after anastomotic vs. handsewn suture closure of the pancreatic stump during DP (Fig 4B, S1 Table). Here, it was evident that enteric or gastric anastomosis of the pancreatic stump was superior to manual suture (OR 0.51, 0.30 to 0.88; *p* = 0.02, Fig 4B), which was even more evident in the comparison of grade B/C PF (OR 0.36, 0.20 to 0.65; *p* = 0.0007, Fig 4B, S1 and S2 Tables). This finding was in line with the results of the only RCT including these techniques.[24] The studies were not heterogeneous in the comparison of anastomosis with stapler (*I*^*2*^ = 0%, Fig 4A, S1 Table), but they were moderately heterogeneous in the anastomosis vs. suture comparison (*I*^*2*^ = 40%, Fig 4B, S1 Table). Furthermore, the funnel plot demonstrated asymmetry in the analysis of anastomosis versus suture (Fig 4).

#### Impact of spleen preservation on fistula rates

**Fig 5:**
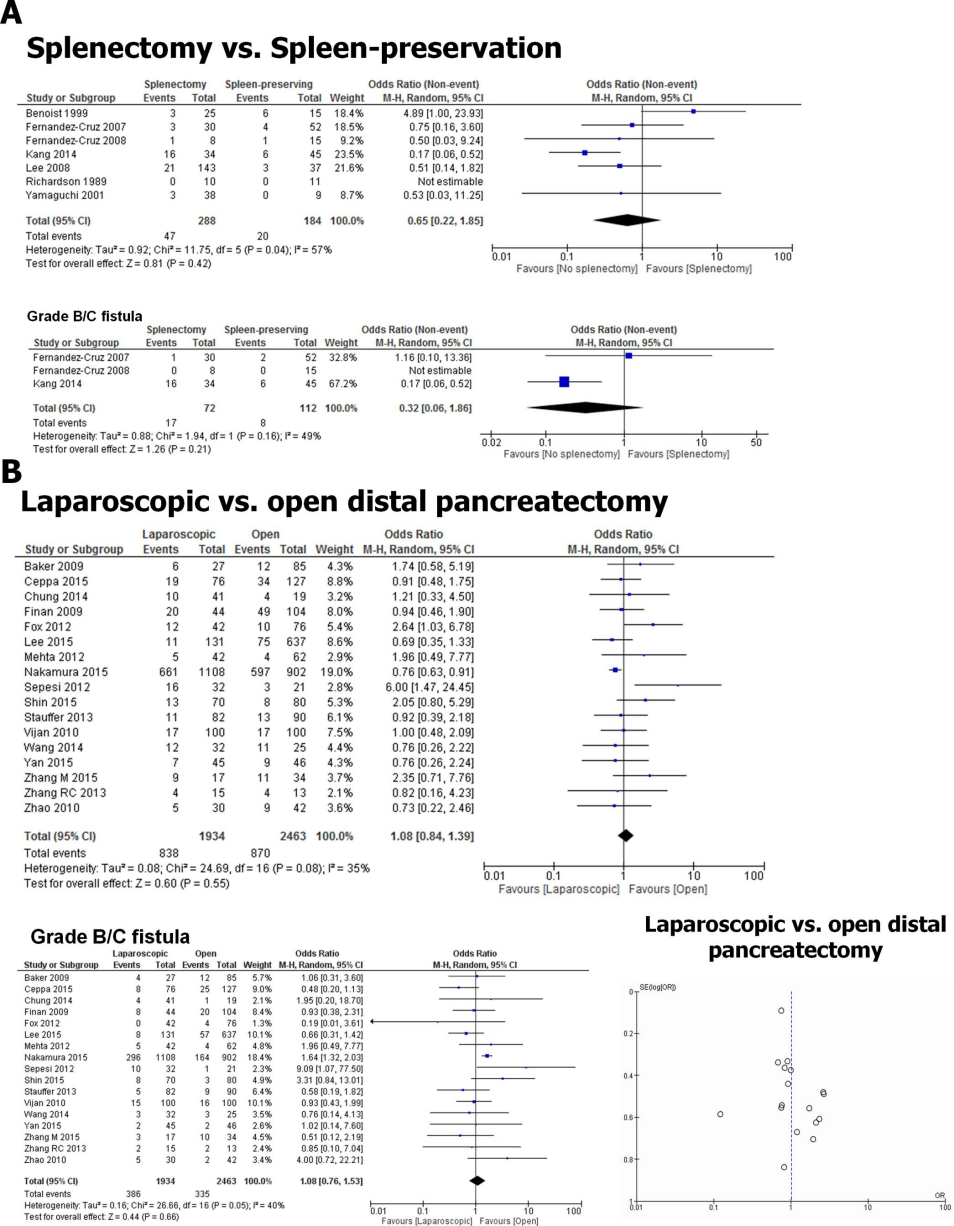
Impact of spleen-preservation and laparoscopic approach on PF rates after DP. **A.** Forrest plot of studies that provided comparative data on overall (upper plot) and clinically relevant (grade B/C, lower plot) PF rates after either spleen-preserving or spleen-resecting DP. **B.** The Forrest plot of studies that compared overall (upper plot) and clinically relevant (grade B/C, lower plot) PF rates after laparoscopic vs. open DP, showing no difference in PF probability after either approach. Bottom right: Funnel plot of the included studies in the comparison of laparoscopic vs. open DP with regard to PF rates. Studies that were associated with high PF rates after laparoscopic DP seem to be lacking.

A systematic review and meta-analysis of the current biomedical data on the impact of spleen preservation on PF formation after DP is not present. Therefore, we extracted data on the PF rates from studies that compared spleen-preserving vs. spleen-resecting variants of DP. Here, the meta-analysis of 7 studies [39–45] (1 prospective, 6 retrospective, S1 Table) with a total of 472 patients showed that spleen-preservation was not associated with a different overall (OR 0.65, 0.22 to 1.85; *p* = 0.42, Fig 5A) or clinically relevant grade B/C PF rate (OR 3.09, 0.54 to 17.84; *p* = 0.21, Fig 5A, S2 Table) when compared to splenectomy. There was high heterogeneity among the included studies (*I*^*2*^ = 57%).

#### Laparoscopic vs. open distal pancreatectomy

Minimally invasive, especially laparoscopic DP is increasingly becoming the mainstay surgical technique for resection of the pancreatic tail [10]. Data on the PF rates after laparoscopic DP in comparison with open DP are scarce [10]. Therefore, we also performed a systematic review of the PF rates after laparoscopic versus open DP. A total of 17 studies [26, 46–61] (4 prospective, 13 retrospective, S1 Table) with 4,389 patients were identified that reported on the fistula rates in simultaneous comparison of both techniques (Fig 5B). Here, there was no overt difference in the overall (OR 1.08, 0.84 to 1.39; *p* = 0.55, Fig 5B) or clinically relevant grade B/C PF rates (OR 1.08, 0.76 to 1.53; *p* = 0.66, Fig 5B, S1 and S2 Tables) after laparoscopic versus open DP. The studies exhibited a low to medium level of heterogeneity (*I*^*2*^ = 35, S1 Table). Especially, based on the plot, studies that specifically reported relatively higher PF rates after laparoscopic DP seemed to be lacking (Fig 5C).

#### Human fibrinogen/thrombin sealant (TachoSil®) and PF rates

**Fig 6:**
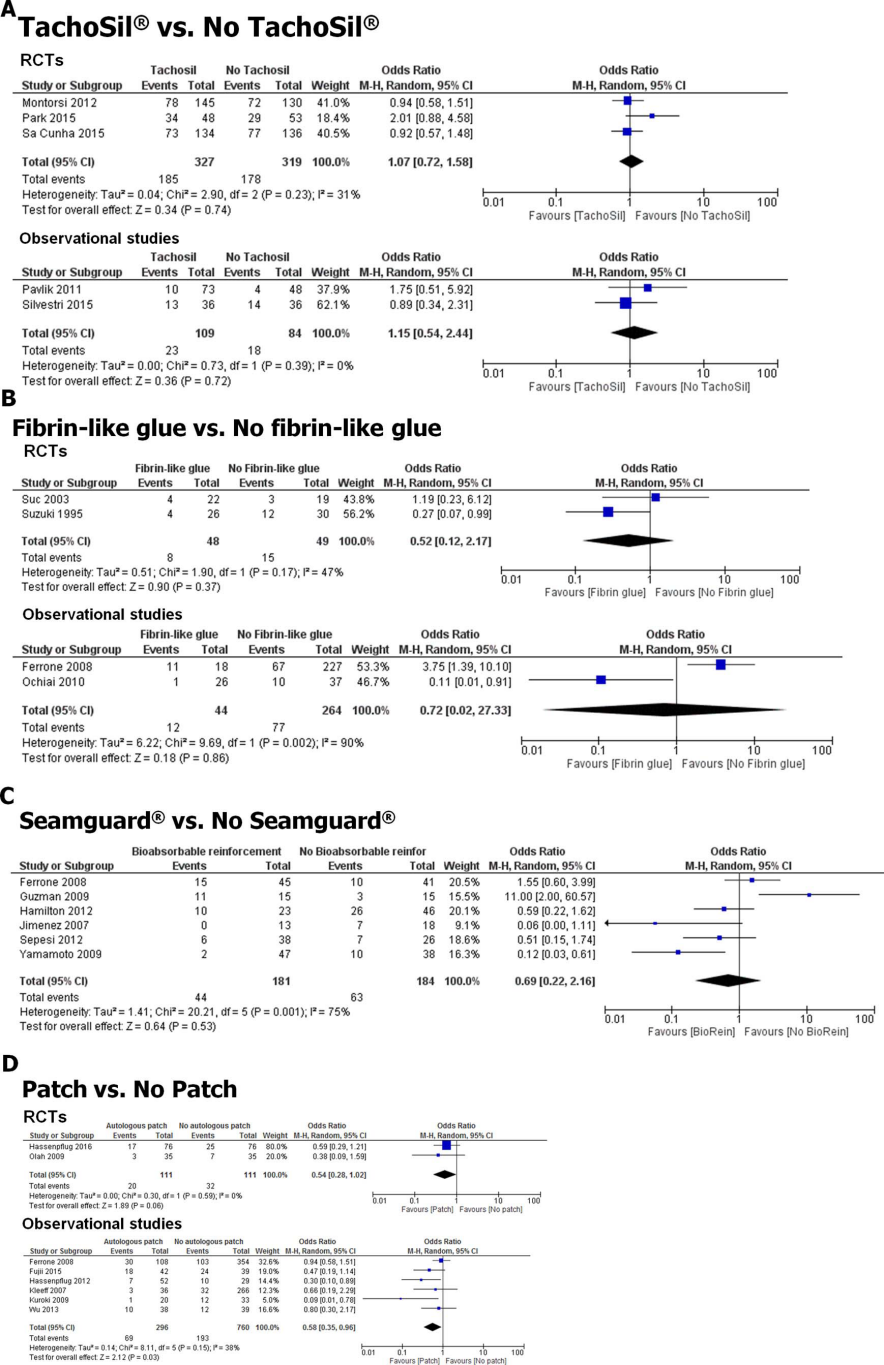
Stump “coverage” techniques and PF rates after DP. **A.** Three RCTs analyzed the effect of TachoSil^®^ application on PF rates, where TachoSil^®^ clearly lacked any benefit. RCTs: randomized controlled trials. **B.** Forrest blot of 2 RCTs and 2 retrospective studies that analyzed the impact of fibrin-glue application on PF rates after DP. **C.** The bioabsorbable staple-line reinforcement Seamguard^®^ did also not relevantly influence PF rates in the five corresponding studies. BioRein: bioabsorbable staple-line reinforcement/Seamguard^®^. **D.** Application of biological, i.e. autologous, seromuscular enteric, omental, falciform ligament, teres hepatis ligament patches was associated with a lower risk of postoperative PF after DP. 95%CI: 95% confidence interval.

In the past two decades, several studies also investigated the potential benefit of fibrinogen-based sealents like TachoSil^®^ in the prevention of PF after DP. Specifically, investigators compared the PF rates after stapler- or suture-based transection of the pancreatic tail and subsequent TachoSil^®^ application. We identified five studies [18, 20, 62–64] (3 RCTs, 2 retrospective, S1 Table) that contained quantitative data from the comparison of TachoSil^®^ vs. no TachoSil^®^ sealing in a total number of 839 patients. Notably, three studies were randomized controlled trials [18, 20, 63] including a total of 646 patients, and there was no heterogeneity between these studies (*I*^*2*^ = 0). The pooled estimate from these studies revealed no preventive effect of TachoSil^®^ application against overall PF formation after DP (OR 1.05; 0.79 to 1.40, *p* = 0.73, Fig 6A) or for reducing clinically relevant grade B/C fistula (OR 0.97; 0.60 to 1.58, *p* = 0.91, S1A Fig, S1 and S2 Tables). In the separate analysis of the RCTs and observational studies, there was also no beneficial effect of TachoSil^®^ on PF rates (RCTs: OR 1.07; 0.72 to 1.58, *p* = 0.74; observational studies: OR 1.15; 0.54 to 2.44, *p* = 0.72, Fig 6A). Due to the small number of included studies, we refrained from the analysis of publication bias.

#### Fibrin-like glues and fistulas

In addition to TachoSil^®^, further fibrin-based compounds have been developed with a “glue-like” feature and tested for their effectiveness in the prevention of PF after DP. We could identify 4 studies [7, 21, 65, 66] with a total of 405 patients, where there were two RCTs and two retrospective case series. The four included studies exhibited a very high heterogeneity (*I*^*2*^ = 80). Here, application of fibrin-like glues was found to have no effect on the overall PF rate (OR 0.68, 0.13 to 3.44; *p* = 0.64, Fig 6B) or on the rate of clinically relevant grade B/C fistula (OR 0.31; 0.07 to 1.34, *p* = 0.12, S1B Fig, S1 and S2 Tables) after DP. When RCTs and observational studies were analyzed separately, there was similarly no beneficial effect of fibrin-like glues on PF rates (RCTs: OR 0.52; 0.12 to 2.17, *p* = 0.37; observational studies: OR 0.72; 0.02 to 27.33, *p* = 0.86, Fig 6A). Publication bias was not analyzed due to the small number of included studies.

#### Bioabsorbable reinforcements

An alternative approach of pancreatic stump closure during DP is the usage of a synthetic, polyglycolic acid-based bioabsorbable staple line reinforcement, e.g. Seamguard^®^. A total of six studies [23, 53, 65, 67–69] (5 retrospective, 1 RCT) including 365 patients analyzed the effect of Seamguard^®^ on the PF rate after DP in simultaneous comparison with omission of such a bioabsorbable staple line reinforcement. In the present meta-analysis, the application of such a staple line reinforcement did not affect the overall PF rate after DP (OR 0.69, 0.22 to 2.16; *p* = 0.53, Fig 6C, 24% in the reinforcement vs. 34% in the no reinforcement group). There was only one study that provided data on the rate of clinically relevant grade B/C fistulas [67], so that a meta-analysis could not be performed for clinically relevant fistulas after Seamguard^®^ application. The included studies exhibited major heterogeneity (*I*^*2*^ = 75%, Fig 6C).

#### Autologous patches

A frequently reported method that has found increasing acceptance in the recent years for the coverage of the pancreatic stump during DP is the placement of autologous tissue patches like the falciform/teres hepatis ligament, seromuscular patches from the jejunum or ileum, or omentum patches. The systematic review of the literature revealed a total of eight studies [2 RCTs [9, 70], 1 prospective [8] and 5 retrospective case series [12, 65, 71–73]] including 1,126 patients with or without autologous coverage of the pancreatic stump. Additional coverage with autologous patches lead to decreased overall PF rates when compared to pancreatic stump closure with no patch (OR 0.60, 0.41 to 0.86; *p* = 0.006, Fig 6D). Importantly, the protective effect of patches was more prominent for the incidence of grade B/C fistula (OR 0.49, 0.30 to 0.78; *p* = 0.003, S1C Fig). Interestingly, the protective effect of autologous patches reached statistical significance only in the meta-analysis of observational studies, but not in the meta-analysis of RCTs (Figs 6D and S3C). There was rather low heterogeneity of data in the included six studies (*I*^*2*^ = 20).

### Institutional experience in pancreatic stump closure during DP and the impact of selective closure of the main pancreatic duct

**Fig 7:**
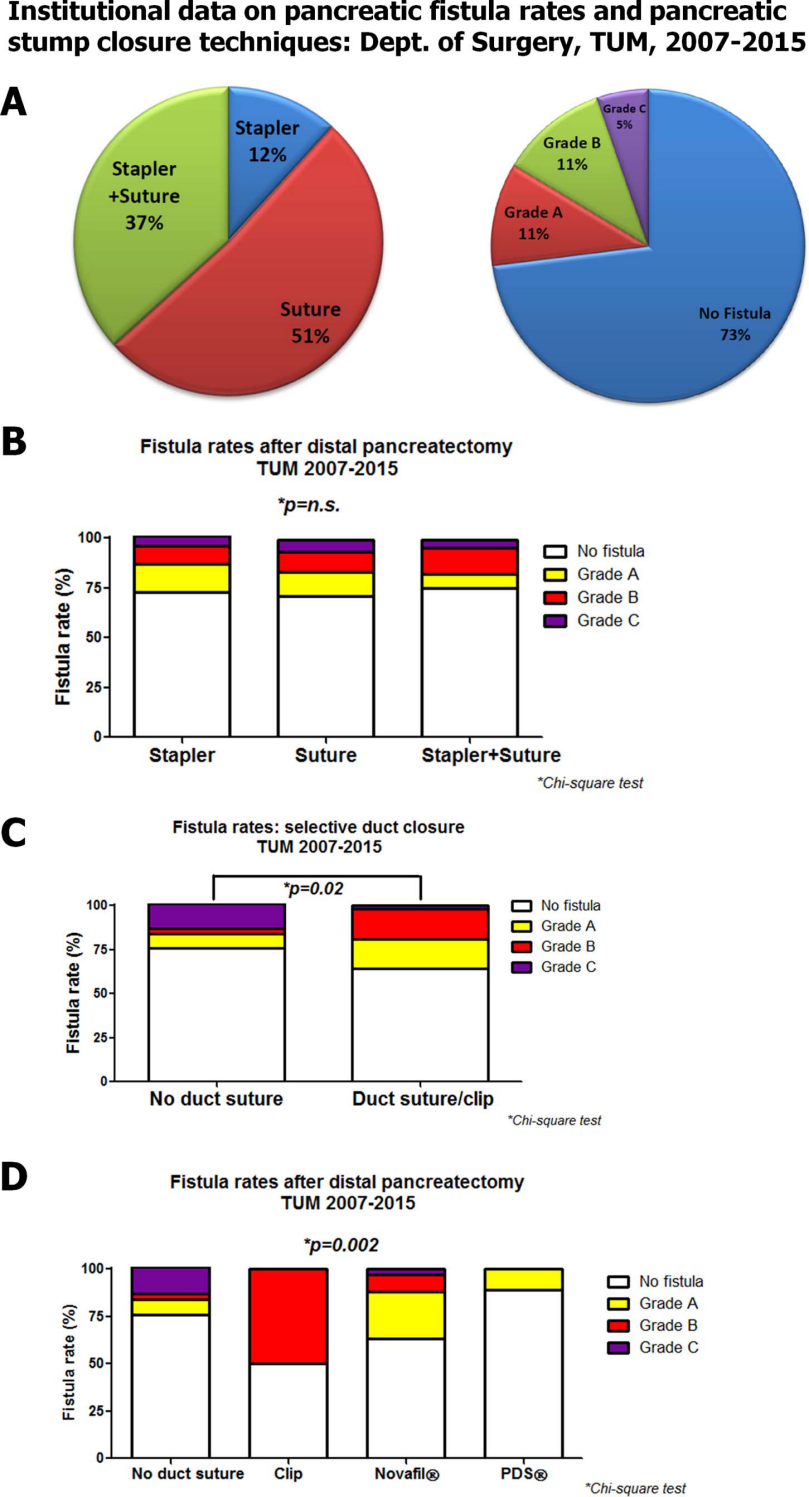
Retrospective analysis of our institutional experience on PF rates and stump closure techniques during DP (Department of Surgery, TU München, 2007–2015). **A.** Three different techniques of stump closure were applied for closure of the pancreatic remnant, where suture closure dominated. The overall PF rate was 27% (Grade A: 11%, Grade 2: 11%, Grade 3: 5% according to the ISGPS definition from 2005 [16]). **B.** The overall PF rate, but also the grade of the PF did not differ between DPs performed via any of the three techniques. n.s.: not significant. **C.** We also analyzed whether targeted, i.e. extra closure of the main pancreatic duct (e.g. via sutures or clipping) prior to suturing of the whole stump during DP influenced PF grade. Indeed, duct suturing or clipping prior to stump suture decreased the proportion of higher grade PF. **D.** The beneficial effect of pancreatic duct closure on reduction of higher grade PF was most obvious for closure with the monofilamentous PDS^®^ sutures.

To compare the results of the current meta-analysis with our own experience, we retrospectively analyzed the incidence of PF in the time period of 2007–2015 at our own institution and classified the PFR according to the ISGPS definition [16]. In the specified period, we performed a total of 188 consecutive DP, using three different stump closure techniques: hand-sewn suture (51% of cases), stapler closure (12%), or the combination of both (37%, Fig 7A, S3 Table). The overall PF rate was 27% (Fig 7A, S3 Table). Here, we detected no difference in the overall PF rates (p = 0.83) or in the severity of PF (p = 0.92) due to either technique (Fig 7B, S3 Table). However, we noticed that during handsewn stump closure, the surgeons chose to either perform a selective clip- or suture closure of the pancreatic duct in 62% of cases, whereas in 38% the pancreatic stump was sutured without previous selective duct closure. Although the overall fistula rate did not differ between “no duct closure” and “duct closure” groups (p = 0.25, S3 Table), the selective duct closure was associated a lower frequency of higher grade, i.e. Grade C, fistulas (Fig 7C, S3 Table). In the comparison of the different duct closure techniques (clipping, Z-shaped suturing of the duct with the monofilamentous Novafil^®^ or PDS^®^), especially PDS^®^ closure led to significant prevention of higher Grade (B or C) PF (p = 0.002, Fig 7D, S3 Table).

Finally, we added our own institutional data to the data of our meta-analysis and re-compared the outcomes. Here, addition of our data did not affect the observed effects in the meta-analysis (S2 and S3 Figs). Indeed, stapler closure remained superior to suture closure for overall fistula rate in the meta-analysis of observational studies including ours (OR 0.72, 0.55 to 0.95; *p* = 0.02, S2A Fig), but again not affecting the Grade B/C fistulas (OR 0.62, 0.35 to 1.11; *p* = 0.11, S2B Fig). The combined stapler+suture closure was again comparable to sole stapler closure after the inclusion of our institutional data with regard to overall PF rate (OR 0.79, 0.56 to 1.13; *p* = 0.20, S3A Fig), and Grade B/C PF rate (OR 1.01, 0.56 to 1.85; *p* = 0.96, S3A Fig). Similarly, the combined stapler+suture closure again tended to lead to lower overall PF rates (OR 0.70, 0.50 to 1.00; *p* = 0.05, S3B Fig), yet not affecting Grade B/C fistula rates (OR 0.79, 0.50 to 1.26; *p* = 0.32, S3B Fig) when compared to suture closure alone.

## Discussion

The present study represents, to our knowledge, the largest meta-analysis of all major stump closure techniques during DP and their associated overall and clinically relevant, i.e. grade B/C PF rates. Our results suggest the superiority of stapler, combined stapler and suture closure, anastomosis of the pancreatic stump into the jejunum or stomach, and autologous patch application when compared to manual suture closure of the pancreatic stump. Furthermore, there seems to be no impact of laparoscopic versus open DP, spleen-preserving vs. spleen-resecting DP, and of sealents like TachoSil®, fibrin-like glues, or the stapler bio-reinforcement Seamguard® on PF rates after DP.

One factor that may affect the outcome of our analyses is the definition of PF. In our meta-analysis, 40 out of 81 studies stuck to the ISGPF definition of pancreatic fistula [16], which is why we could perform additional subanalyses with the clinically relevant Grade B/C. The majority of studies that did not state to have stuck to the ISGPF definition of pancreatic fistula, represent older studies, but even in these studies, pancreatic fistula definition was mostly based on measurements of amylase in the drainage and its comparison with the serum amylase. In some studies, the authors additionally used definitions such as radiological evidence of leak from the pancreas [7]. Thus, although only half of all included studies stuck to the ISGPF definition, most other, the rather older studies, frequently made use of the biochemical measurement of amylase in the drain fluid for the definition of pancreatic fistula. Therefore, we believe that due to the widespread measurement of amylase in the drain fluid, the included studies in our meta-analysis are comparable. Furthermore, the timing of drain placement, and the frequency of routine drain placement during surgery, are, also in our view, critical determinants of the natural course of PF In the studies included in our meta-analysis, most studies made use of drains to measure amylase in the secreted fluid; however, several studies did not contain specific information on the exact timing of drains. For example, several studies do not explicitly mention whether drains were placed during surgery or by an interventional radiologist postoperatively. As we stated above, most studies, though, made use of amylase measurement in the drain fluid in the early postoperative days: thus, we believe that the impact of drains on the natural course of pancreatic fistulae should also be comparable among most of the included studies.

Any interpretation of our results should consider the quality and the specific characteristics of the included studies. Zhang et al.’s [11] and our meta-analysis identified the superiority of the stapler versus manual suture of the stump. The majority of all included studies was retrospective and included only small numbers of patients. However, even among the two available RCTs that compared stapler with suture during DP, Bassi et al. [24] included a total of only 29 patients (with 15 in the suture and 14 patients in the stapler group). Moreover, the isolated analysis of the two available RCTs yet revealed no major difference in the fistula rates by either technique both in our and Zhang et al.’s meta-analysis [11]. So far, the strongest evidence regarding the comparison of stapler versus suture comes from the DISPACT trial [3], which did not demonstrate any difference in PF rates after DP via stapler or suture at all. Hence, the “calculated” superiority of stapler versus suturing may not reflect the clinical reality, and, for now, it seems to be more correct to rather assume no difference in PF rates after DP via stapler or suture closure. This interpretation would go in line with the conclusion of the most recent Cochrane review by Probst et al. that specifically focused on these two techniques.[74] Therefore, only an additional multicentre RCT may shed light and enable a genuine conclusion on this still unclear issue.

In the comparison of the stapler-suture combination with either technique alone, the number of included studies was limited to six or seven, respectively, and the total number of patients in each arm varied quite largely from 15 to 130. A major drawback of these exclusively retrospective studies is that it is not clear when and why the surgeons preferred combined stapler-suture closure compared to isolated suture or stapler closure. In these seven studies, one cannot exclude that the surgeons preferred combination closure in more “high-risk” cases (e.g. based on pancreatic duct diameter or pancreatic stiffness). Furthermore, one cannot extract conclusive information on whether the authors, while suturing the pancreatic stump, performed a selective suture/ligation/clipping of the main duct. Due to the lack of a RCT including a technical combination arm (i.e., stapler and suture versus each method alone), it seems that the choice for combined stapler-suture closure during DP is currently completely left to the personal preference of the surgeon, and based on current evidence, not have sufficiently beneficial effect on PF rates.

In the present meta-analysis, anastomosis of the pancreatic stump revealed a statistically beneficial effect on PF rates when compared to suture during DP. This effect is generated by the weight of two studies (Kleeff et al. [12] and Wellner et al. [33]). In both studies, the background for the preference of anastomosis over suturing has not been explained, so the presence of patient selection bias can also not be excluded. Rationally, drainage of the stump into the intestine and coverage of the stump by protective intestinal serosa, may lead to lower PF rates. The pancreaticoduodenectomy/PD also incorporates an pancreatico-enteric anastomosis, which is associated with lower average PF rates (16% for PD vs. approximately 31% for DP) [75]. However, despite this logical explanation, Kawai et al. [22] have recently shown in a RCT setting, that anastomosis may not always be superior to other closure techniques. Therefore, before recommending any routine consideration of pancreatic anastomosis during DP, we should await the results of two RCTs from Japan that currently investigate the PF after pancreatico-jejunostomy or pancreatico-gastrostomy [30].

The present meta-analysis contributed to the accumulating evidence on the comparability of postoperative morbidity after laparoscopic versus open DP [10, 76]. We could include one very recent prospective multi-centre study with 91 patients [58], and another new large-scale multi-centre study with 2,010 patients [52]. In line with the recent meta-analyses [10, 76], our current meta-analysis with 4,186 patients confirmed the comparable PF rates after laparoscopic versus open DP. The careful consideration of the included studies reveals a huge variation in the included number of subjects (e.g. approximately 1,000 per arm in the Nakamura study [52] vs. down to 15 per arm [59]). Furthermore, publication bias toward reporting of studies with laparoscopic lower PF rates was evident. This bias is likely to result from better patient selection in the laparoscopic arms, from the performance of laparoscopic DP by selected surgeons with particular laparoscopic expertise, or to potential unpublished results demonstrating higher PF rates after laparoscopic DP. There is yet no single RCT that compared the perioperative morbidity and PF rates after laparoscopic versus open DP. In the presence of publication bias and lack of high-grade evidence, we feel that one should refrain from reaching a conclusion on the laparoscopic or open DP-associated PF rates.

In the comparison of the “synthetic” biosealants, the evidence from studies on TachoSil^®^ is certainly strong, since three of the included five studies represented RCTs with at least 48 patients per arm [18, 20, 63]. Despite the lack of comparably strong evidence from the available studies on other sealants like fibrin-glue or Seamguard^®^, these techniques altogether do not seem to contribute toward lower PF rates. Especially in the non-randomized studies that investigated these sealants, the investigators did not specify the reasons for applying these agents in the respective cases.

Our findings on the fistula-reducing effect of autologous tissue patches during DP require further attention. Despite the much higher number of patients who received no autologous tissue patch in this meta-analysis, the data from the two RCTs point to a potential beneficial impact of such patches. In the recent DISCOVER trial [70, 77] that involved coverage of the pancreatic stump with a teres hepatis ligament patch, the overall clinically relevant grade B/C fistula rate was not reduced. However, the investigators found lower rates of re-interventions, re-operations, and re-admissions. Thus, although it may not reduce the overall fistula rate, such autologous patches seem to at least alleviate the clinical course of grade B/C PF.

Based on our institutional data, the selective primarily closure of the main pancreatic duct may inhibit formation of Grade C PF. In the literature, we could not identify studies that compared the targeted closure of the main pancreatic duct with sutures or clips, to the omission of such a duct closure during hand-sewn closure of the pancreatic stump. Pathophysiologically, if PF emerges due to the leakage from the pancreatic duct at the stump, preventive closure of the main duct, may indeed enable lower-secretion fistulas.

In conclusion, the present meta-analysis showed that, despite the statistical superiority of some techniques for preventing PF during DP, the biomedical evidence for a true benefit of the analyzed techniques seems not to be strong enough. Furthermore, the detected differences in the PF “risks” attributable to either technique are small. Still, indicators of a potentially genuine benefit of autologous patch closure of the pancreatic stump exist. Until the advent of well-designed, bias-free, high-powered studies, surgeons may consider combining their expertise in any stump closure technique with the herein reported benefits of patch application or selective duct closure.

## Supporting information

S1 Fig A. Forrest plot of studies that compared the rates of clinically relevant pancreatic fistula (PF) in the presence or absence of TachoSil® on the pancreatic stump. B. Fibrin application does not affect the incidence of grade B/C fistulas after distal pancreatectomy. C. In line with the overall PF rate (Fig 6D), application of autologous patches, e.g. falciform patch, on the pancreatic stump reduced the rate of clinically relevant grade B/C fistulas.
(TIF)

S2 Fig Meta-analysis combining pancreatic fistula (PF) rates from our data (Tieftrunk et al.) with the previously published data. Forrest plot of studies that compared the overall (panel A) and clinically relevant (grade B/C, lower plot, panel B) PF rates after DP with stapler versus suture closure of the pancreatic stump. 95%CI: 95% confidence interval.
(TIF)

S3 Fig Meta-analysis combining pancreatic fistula (PF) rates from our data (Tieftrunk et al.) with the previously published data. **A.** Forrest plot of studies that compared the overall and clinically relevant (grade B/C, lower plot) PF rates after DP with combined stapler and suture closure versus isolated stapler of the pancreatic stump. **B.** Forrest plot of studies that compared the overall and clinically relevant (grade B/C, lower plot) PF rates after DP with combined stapler and suture closure versus isolated suture of the pancreatic stump. 95%CI: 95% confidence interval.
(TIF)

S1 Table Overview of the subanalyses, the pooled odds ratios (ORs) for fistula formation, and the level of heterogeneity. RCT: randomized controlled trials. Obs.: observational studies.
(DOC)

S2 Table Overview of the pooled odds ratios (ORs) for formation of clinically relevant, i.e. Grade B/C fistula (according to the definition of the International Study Group on Pancreatic Surgery/ISGPS, Bassi et al., *Surgery* 2005). (DOC)

S3 Table Institutional data (Department of Surgery, Technische Universität München, 2007–2015) on the impact of stump closure technique (upper half) and of the selective duct closure technique on the pancreatic fistula rate after distal pancreatectomy. The International Study Group on Pancreatic Surgery (ISGPS) definition of pancreatic fistula (Grade A, B or C; Bassi et al., *Surgery* 2005) was applied.
(DOC)

S1 Checklist PRISMA 2009 checklist. The PRISMA guidelines were considered in the design of the present study.
(PDF)
