# Supplementary figures and images for: Pancreatic stump closure techniques and pancreatic fistula formation after distal pancreatectomy: Meta-analysis and single-center experience
Source: PLoS One. 2018 Jun 13;13(6):e0197553. doi: 10.1371/journal.pone.0197553 (PMC5999073; doi:10.1371/journal.pone.0197553)

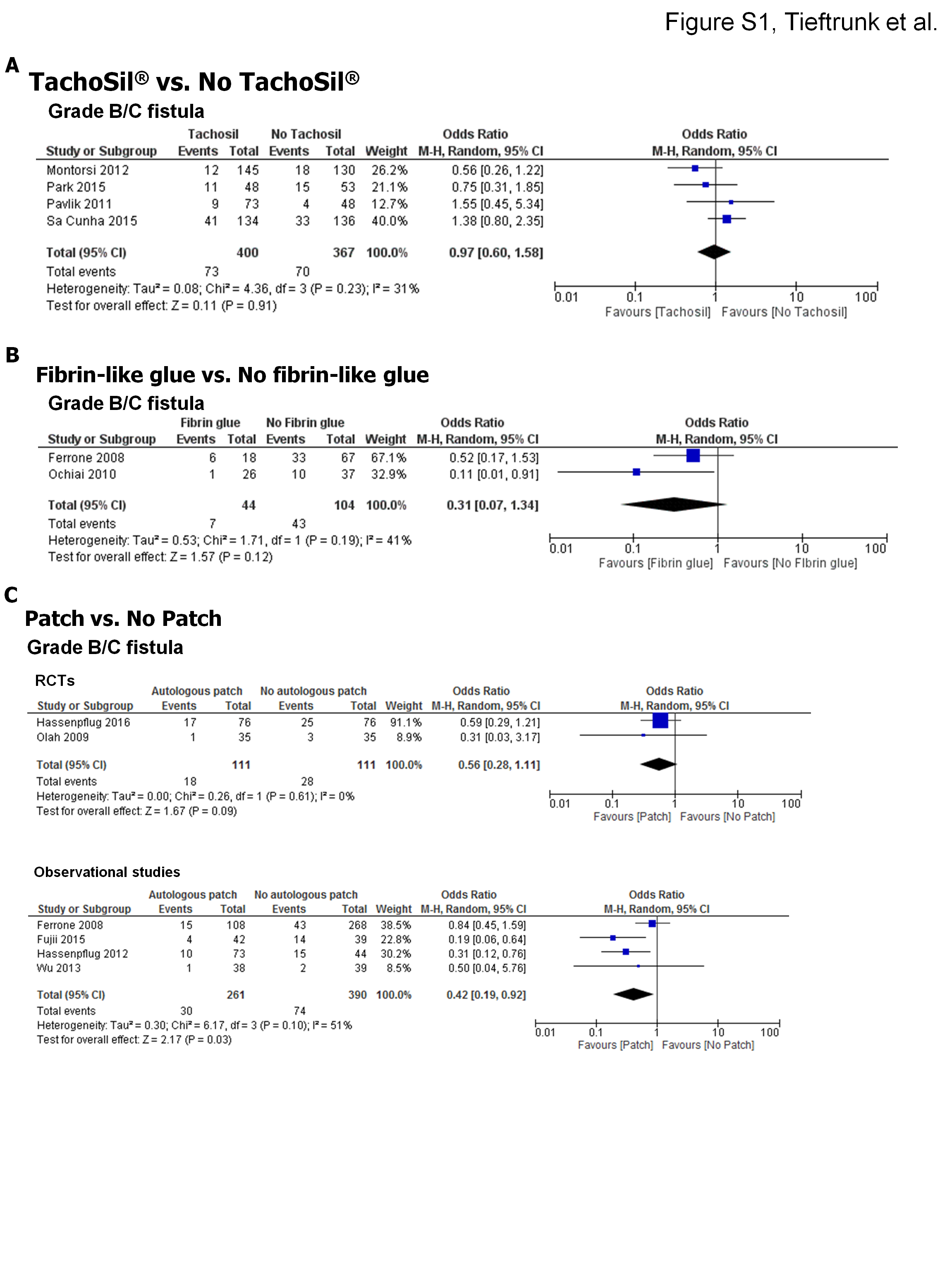

Supplement: S1 Fig — A. Forrest plot of studies that compared the rates of clinically relevant pancreatic fistula (PF) in the presence or absence of TachoSil® on the pancreatic stump. B. Fibrin application does not affect the incidence of grade B/C fistulas after distal pancreatectomy. C. In line with the overall PF rate (Fig 6D), application of autologous patches, e.g. falciform patch, on the pancreatic stump reduced the rate of clinically relevant grade B/C fistulas. (TIF) [file pone.0197553.s001.TIF]

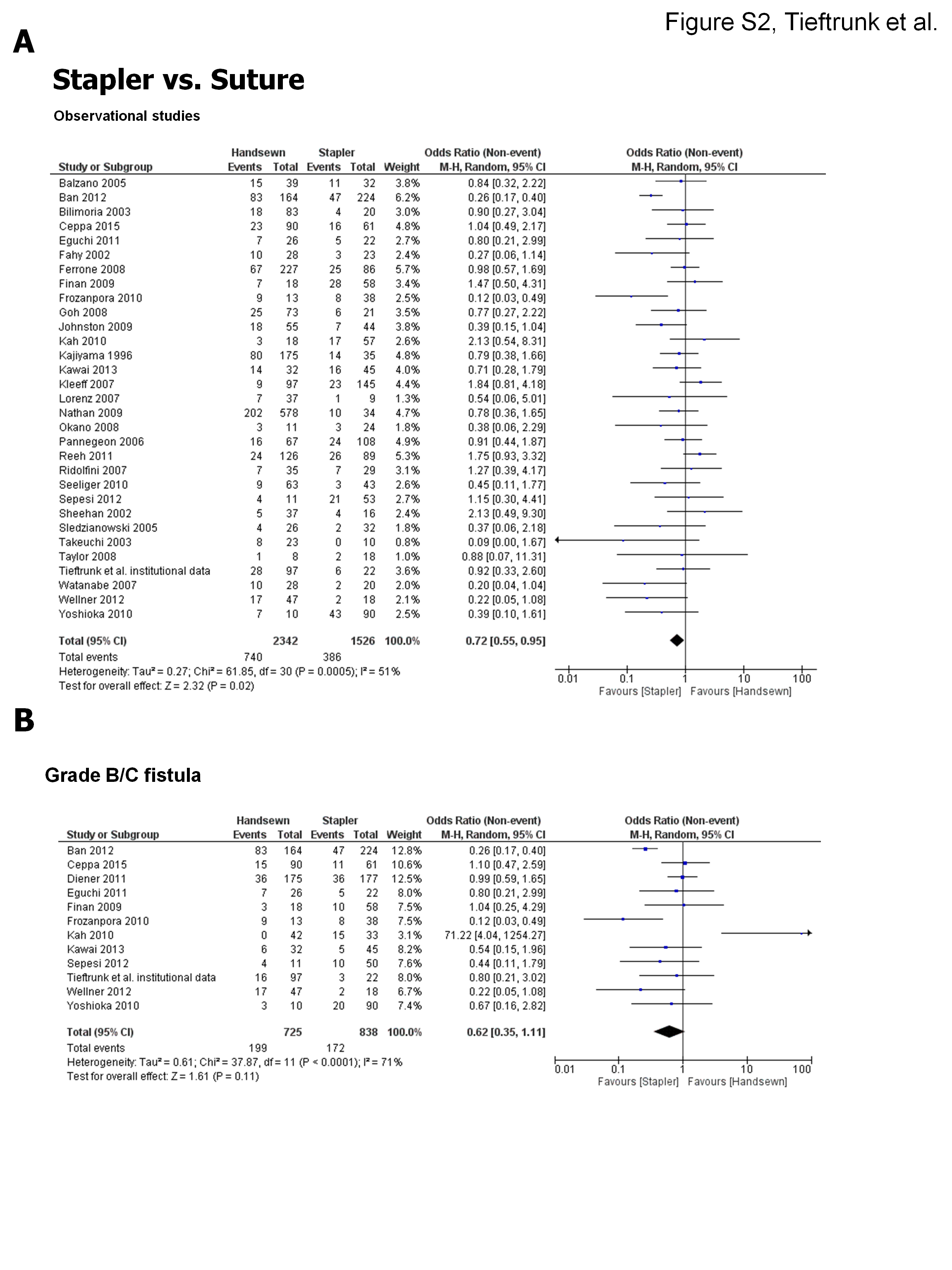

Supplement: S2 Fig — Forrest plot of studies that compared the overall (panel A) and clinically relevant (grade B/C, lower plot, panel B) PF rates after DP with stapler versus suture closure of the pancreatic stump. 95%CI: 95% confidence interval. (TIF) [file pone.0197553.s002.TIF]

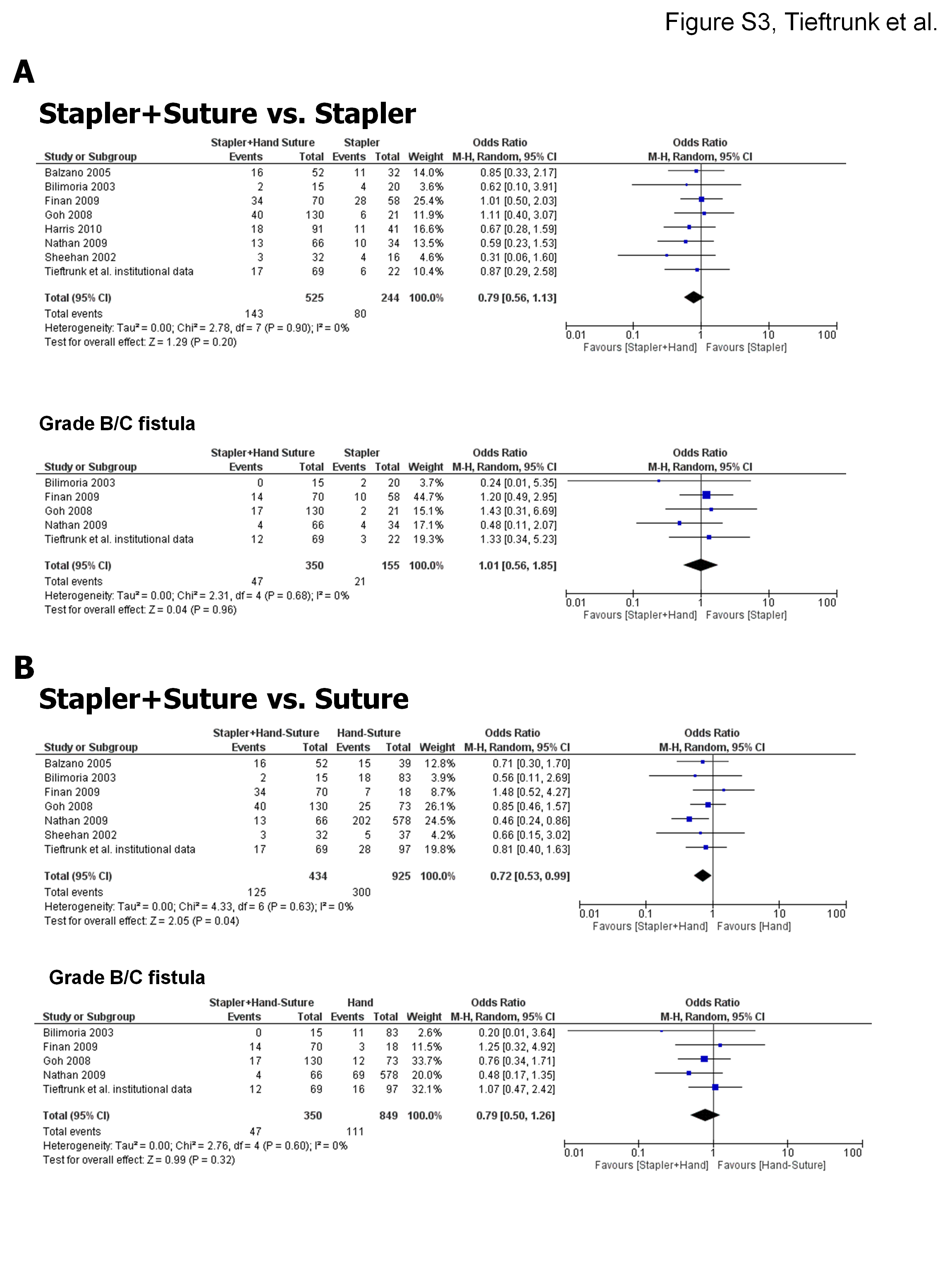

Supplement: S3 Fig — A. Forrest plot of studies that compared the overall and clinically relevant (grade B/C, lower plot) PF rates after DP with combined stapler and suture closure versus isolated stapler of the pancreatic stump. B. Forrest plot of studies that compared the overall and clinically relevant (grade B/C, lower plot) PF rates after DP with combined stapler and suture closure versus isolated suture of the pancreatic stump. 95%CI: 95% confidence interval. (TIF) [file pone.0197553.s003.TIF]
