# Supplementary material for: Pancreatic stump closure techniques and pancreatic fistula formation after distal pancreatectomy: Meta-analysis and single-center experience
Source: PLoS One. 2018 Jun 13;13(6):e0197553. doi: 10.1371/journal.pone.0197553 (PMC5999073; doi:10.1371/journal.pone.0197553)
Supplement: S1 Table — RCT: randomized controlled trials. Obs.: observational studies. (DOC) [file pone.0197553.s004.doc]

**S1 Table.** **Overview of the subanalyses, the pooled odds ratios (ORs) for fistula formation, and the level of heterogeneity.** RCT: randomized controlled trials. Obs.: observational studies.

| **Technical comparison** |  | **No. of patients** | **No. of studies** | **RCT** | **Prospective** | **Retrospective** | **Events** | **OR** | **95% c.i.** | **Z** | ***p*** | **Chi^2^** | ***p*** | ***I*^2^ (%)** |
| --- | --- | --- | --- | --- | --- | --- | --- | --- | --- | --- | --- | --- | --- | --- |
| Stapler vs. Suture |  | 4130 | 32 | 2 | 2 | 28 | Stapler: 48 of 1695  Suture: 766 of 2435 | 0.73 | 0.56 to 0.95 | 2.31 | 0.02 | 66.69 | 0.0002 | 54 |
|  | *RCTs* | *381* | *2* | *2* | *0* |  | *Stapler: 58 of 191*  *Suture: 54 of 190* | *0.87* | *0.30 to 2.55* | *0.25* | *0.80* | *1.73* | *0.19* | *54* |
|  | *Obs.* | *3749* | *30* | *0* | *2* | *28* | *Stapler: 380 of 1504*  *Suture: 712 of 2245* | *1.40* | *1.05 to 1.86* | *2.33* | *0.02* | *61.60* | *0.0004* | *53* |
| Stapler+Suture vs. Stapler |  | 678 | 7 | 0 | 0 | 7 | Combination: 126 of 456  Stapler: 74 of 222 | 0.79 | 0.54 to 1.14 | 1.27 | 0.20 | 2.75 | 0.84 | 0 |
| Stapler+Suture vs. Suture |  | 1193 | 6 | 0 | 0 | 6 | Combination: 108 of 365  Stapler: 272 of 828 | 0.70 | 0.50 to 1.00 | 1.99 | 0.05 | 4.21 | 0.52 | 0 |
| Anastomosis vs. Stapler |  | 655 | 8 | 2 | 1 | 5 | Anastomosis: 59 of 241  Stapler: 94 of 414 | 1.00 | 0.65 to 1.53 | 0.01 | 0.99 | 4.77 | 0.69 | 0 |
|  | *RCTs* | *151* | *2* | *2* | *0* |  | *Anastomosis: 25 of 76*  *Stapler: 25 of 75* | *0.98* | *0.49 to 1.97* | *0.06* | *0.96* | *0.37* | *0.54* | *0* |
|  | *Obs.* | *504* | *6* | *0* | *1* | *5* | *Anastomosis: 34 of 165*  *Stapler: 69 of 339* | *1.01* | *0.58 to 1.74* | *0.03* | *0.97* | *4.47* | *0.48* | *0* |
| Anastomosis vs. Suture |  | 1645 | 14 | 1 | 3 | 10 | Anastomosis: 54 of 389  Suture: 354 of 1256 | 0.51 | 0.30 to 0.88 | 2.40 | 0.02 | 21.67 | 0.86 | 40 |
| Splenectomy vs. Spleen-preservation |  | 472 | 7 | 0 | 1 | 6 | Splenectomy: 47 of 288  Spleen-preservation: 20 of 184 | 0.65 | 0.22 to 1.85 | 0.81 | 0.42 | 11.75 | 0.04 | 57 |
| Laparoscopic vs. open |  | 4397 | 17 | 0 | 4 | 13 | Laparoscopic: 838 of 1934  Open: 870 of 2463 | 1.08 | 0.84 to 1.39 | 0.60 | 0.55 | 24.69 | 0.08 | 51 |
| TachoSil® vs. No TachoSil® |  | 839 | 5 | 3 | 0 | 2 | TachoSil®: 208 of 436  No TachoSil®: 196 of 403 | 1.05 | 0.79 to 1.40 | 0.35 | 0.74 | 3.69 | 0.45 | 0 |
|  | *RCTs* | *646* | *3* | *3* | *0* | *0* | *TachoSil®: 185 of 327*  *No TachoSil®: 178 of 319* | *1.07* | *0.72 to 1.58* | *0.34* | *0.74* | *2.90* | *0.23* | *31* |
|  | *Obs.* | *839* | *2* | *0* | *0* | *2* | *TachoSil®: 23 of 109*  *No TachoSil®: 18 of 84* | *1.15* | *0.54 to 2.44* | *0.36* | *0.72* | *0.73* | *0.39* | *0* |
| Fibrin-glue vs. No fibrin-glue |  | 546 | 4 | 2 | 0 | 2 | Fibrin-glue: 33 of 172  No fibrin-glue: 101 of 374 | 0.68 | 0.13 to 3.44 | 0.47 | 0.64 | 15.22 | 0.002 | 80 |
|  | *RCTs* | *238* | *2* | *2* | *0* | *0* | *Fibrin-glue: 21 of 128*  *No fibrin-glue: 101 of 374* | *0.52* | *0.12 to 2.17* | *0.70* | *0.37* | *3.37* | *0.07* | *70* |
|  | *Obs.* | *546* | *4* | *2* | *0* | *2* | *Fibrin-glue: 12 of 44*  *No fibrin-glue: 77 of 264* | *0.72* | *0.02 to 27.33* | *0.18* | *0.86* | *9.69* | *0.002* | *90* |
| Seamguard® vs. No Seamguard® |  | 365 | 6 | 1 | 0 | 5 | Seamguard®: 44 of 181  No Seamguard®: 63 of 184 | 0.69 | 0.22 to 2.16 | 0.64 | 0.53 | 20.21 | 0.001 | 75 |
| Patch vs. No Patch |  | 1278 | 8 | 2 | 1 | 5 | Patch: 89 of 407  No Patch: 225 of 871 | 0.60 | 0.41 to 0.86 | 2.76 | 0.006 | 8.78 | 0.27 | 20 |
|  | *RCTs* | *238* | *2* | *2* | *0* | *0* | *Patch: 20 of 111*  *No patch: 32 of 111* | *0.54* | *0.28 to 1.02* | *1.89* | *0.06* | *0.30* | *0.59* | *0* |
|  | *Obs.* | *546* | *6* | *2* | *0* | *2* | *Patch: 69 of 296*  *No patch: 193 of 760* | *0.58* | *0.35 to 0.96* | *2.12* | *0.03* | *8.11* | *0.15* | *38* |
