# Supplementary material for: Pancreatic stump closure techniques and pancreatic fistula formation after distal pancreatectomy: Meta-analysis and single-center experience
Source: PLoS One. 2018 Jun 13;13(6):e0197553. doi: 10.1371/journal.pone.0197553 (PMC5999073; doi:10.1371/journal.pone.0197553)
Supplement: S2 Table — (DOC) [file pone.0197553.s005.doc]

**S2 Table.** **Overview of the pooled odds ratios (ORs) for formation of clinically relevant, i.e. Grade B/C fistula** (according to the definition of the International Study Group on Pancreatic Surgery/ISGPS, Bassi et al., *Surgery* 2005)

| **Technical comparison** |  | **No. of patients** | **No. of studies** | **RCT** | **Observational** | **Events** | **OR** | **95% c.i.** | **Z** | ***p*** | **Chi^2^** | ***p*** | ***I*^2^ (%)** |
| --- | --- | --- | --- | --- | --- | --- | --- | --- | --- | --- | --- | --- | --- |
| Stapler vs. Suture |  | 1444 | 11 | 1 | 10 | Stapler: 169 of 816  Suture: 183 of 628 | 0.61 | 0.33 to 1.14 | 1.55 | 0.12 | 37.55 | <0.0001 | 73 |
| Stapler+Suture vs. Stapler |  | 414 | 4 | 0 | 4 | Combination: 35 of 281  Stapler: 18 of 133 | 0.95 | 0.49 to 1.85 | 0.15 | 0.88 | 2.12 | 0.55 | 0 |
| Stapler+Suture vs. Suture |  | 1033 | 4 | 0 | 4 | Combination: 35 of 281  Stapler: 95 of 752 | 0.69 | 0.39 to 1.21 | 1.30 | 0.19 | 4.21 | 0.57 | 0 |
| Anastomosis vs. Stapler |  | 266 | 4 | 1 | 3 | Anastomosis: 16 of 132  Stapler: 18 of 134 | 0.76 | 0.34 to 1.71 | 0.67 | 0.51 | 1.56 | 0.67 | 0 |
| Anastomosis vs. Suture |  | 1118 | 8 | 0 | 8 | Anastomosis: 16 of 186  Suture: 136 of 932 | 0.36 | 0.20 to 0.65 | 3.39 | 0.0007 | 1.79 | 0.97 | 0 |
| Splenectomy vs. Spleen-preservation |  | 184 | 3 | 0 | 3 | Splenectomy: 17 of 72  Spleen-preservation: 8 of 112 | 3.09 | 0.54 to 17.84 | 1.26 | 0.21 | 1.94 | 0.16 | 49 |
| Laparoscopic vs. open |  | 4389 | 17 | 0 | 4 | Laparoscopic: 386 of 1934  Open: 335 of 2463 | 1.08 | 0.76 to 1.53 | 0.44 | 0.66 | 26.66 | 0.05 | 40 |
| TachoSil® vs. No TachoSil® |  | 767 | 4 | 3 | 1 | TachoSil®: 73 of 400  No TachoSil®: 70 of 367 | 0.97 | 0.60 to 1.58 | 0.11 | 0.91 | 4.36 | 0.23 | 31 |
| Fibrin-glue vs. No fibrin-glue |  | 148 | 2 | 0 | 2 | Fibrin-glue: 7 of 44  No fibrin-glue: 43 of 104 | 0.31 | 0.07 to 1.34 | 1.57 | 0.12 | 1.71 | 0.19 | 41 |
| Patch vs. No Patch |  | 873 | 6 | 2 | 4 | Patch: 48 of 372  No Patch: 102 of 501 | 0.49 | 0.30 to 0.78 | 2.96 | 0.003 | 6.48 | 0.26 | 23 |
