# Supplementary material for: Pancreatic stump closure techniques and pancreatic fistula formation after distal pancreatectomy: Meta-analysis and single-center experience
Source: PLoS One. 2018 Jun 13;13(6):e0197553. doi: 10.1371/journal.pone.0197553 (PMC5999073; doi:10.1371/journal.pone.0197553)
Supplement: S3 Table — The International Study Group on Pancreatic Surgery (ISGPS) definition of pancreatic fistula (Grade A, B or C; Bassi et al., Surgery 2005) was applied. (DOC) [file pone.0197553.s006.doc]

**S3 Table.** **Institutional data (Department of Surgery, Technische Universität München, 2007-2015) on the impact of stump closure technique (upper half) and of the selective duct closure technique on the pancreatic fistula rate after distal pancreatectomy.** The International Study Group on Pancreatic Surgery (ISGPS) definition of pancreatic fistula (Grade A, B or C; Bassi et al., *Surgery* 2005) was applied.

|  |  |  |  |  | **Chi^2^** | **p-value** |
| --- | --- | --- | --- | --- | --- | --- |
| **Stump closure (n=188/100%)** | No fistula  (n=137/73%) | Fistula  (n=51/27%) | | | 0.36 | 0.83 |
|  |  | *Grade A (11%)* | *Grade B (11%)* | *Grade C (5%)* | 1.96 | 0.92 |
| Stapler (n=22/12%) | 16 (73%) | 3 (14%) | 2 (9%) | 1 (4%) |  |  |
| Suture (n=97/51%) | 69 (71%) | 12 (13%) | 10 (10%) | 6 (6%) |  |  |
| Stapler+Suture (n=69/37%) | 52 (76%) | 5 (7%) | 9 (13%) | 3 (4%) |  |  |
|  |  |  |  |  |  |  |
| **Selective duct management (n=90/100%)** | No fistula (n=62/69%) | Fistula  (n=28/31%) | | | 1.35 | 0.25 |
|  |  | *Grade A (n=13%)* | *Grade B (11%)* | *Grade C (7%)* | 10.12 | ***0.02*** |
| None (n=37/41%) | 28 (73%) | 3 (8%) | 1 (3%) | 5 (14%) |  |  |
| Clip (n=12/13%) | 6 (50%) | 0 (0%) | 6 (50%) | 0 (0%) |  |  |
| Novafil® (n=32/36%) | 20 (63%) | 8 (25%) | 3 (9%) | 1 (3%) |  |  |
| PDS® (n=9/10%) | 8 (89%) | 1 (11%) | 0 (0%) | 0 (0%) |  |  |
